# Prognostication Following Transcatheter Edge-to-Edge Mitral Valve Repair Using Combined Echocardiography-Derived Velocity Time Integral Ratio and Artificial Intelligence Applied to Electrocardiogram

**DOI:** 10.3390/jpm15080371

**Published:** 2025-08-13

**Authors:** Nadera N. Bismee, Isabel G. Scalia, Mohammed Tiseer Abbas, Juan M. Farina, Milagros Pereyra Pietri, Kamal Awad, Nima Baba Ali, Niloofar Javadi, Sogol Attaripour Esfahani, Hesham Sheashaa, Omar H. Ibrahim, Fatmaelzahraa E. Abdelfattah, F. David Fortuin, Steven J. Lester, John P. Sweeney, Chadi Ayoub, Reza Arsanjani

**Affiliations:** Department of Cardiovascular Diseases, Mayo Clinic, Phoenix, AZ 85054, USA; bismee.naderanaquib@mayo.edu (N.N.B.); abbas.mohammedtiseer@mayo.edu (M.T.A.); farina.juanmaria@mayo.edu (J.M.F.); pereyra.milagros@mayo.edu (M.P.P.); awad.kamal@mayo.edu (K.A.); babaali.nima@mayo.edu (N.B.A.); javadi.niloofar@mayo.edu (N.J.); sheashaa.hesham@mayo.edu (H.S.); ibrahim.omar3@mayo.edu (O.H.I.); abdelfattah.fatmaelzahraa@mayo.edu (F.E.A.); fortuin.david@mayo.edu (F.D.F.); lester.steven@mayo.edu (S.J.L.); sweeney.john3@mayo.edu (J.P.S.); ayoub.chadi@mayo.edu (C.A.)

**Keywords:** ECG-AI, mitral valve transcatheter edge-to-edge repair, mitral valve regurgitation, VTI ratio

## Abstract

**Introduction**: Mitral valve transcatheter edge-to-edge repair (M-TEER) has emerged as a minimally invasive option for high-risk surgical candidates with severe and symptomatic mitral regurgitation (MR), but post-procedure residual mitral valve (MV) dysfunction remains a significant concern. This study evaluates the clinical utility of combining artificial intelligence applied to electrocardiograms (ECG-AI) for diastolic dysfunction (DD) grading and the echocardiography-derived velocity time integral of the MV and left ventricular outflow tract ratio (VTI_MV_/_LVOT_) in predicting prognosis in patients post-M-TEER. **Methods**: A retrospective analysis of patients who underwent M-TEER between 2014 and 2021 was conducted. Patients were categorized based on VTI_MV/LVOT_ and ECG-AI scores into three groups: both normal parameters, either abnormal parameter, or both abnormal parameters to compare outcomes (mortality, major adverse cardiovascular events [MACE], and the need for subsequent MV reintervention) using Kaplan–Meier analysis, multivariable Cox regression models, and net reclassification improvement. **Results**: Overall, 250 patients were included; the median age was 79.5 (IQR: 73.1, 84.6) and 66.4% were male. The combined abnormal VTI_MV/LVOT_ (≥2.5) and ECG-AI score for DD (>1) was associated with higher risk of one-year mortality (adjusted HR: 4.56 [1.04–19.89], *p* = 0.044) and MACE (adjusted HR: 3.72 [1.09–12.72], *p* = 0.037) compared to patients with both normal parameters. **Conclusions**: This study highlights the potential additive value of integrating VTI_MV/LVOT_ and ECG-AI scores as a prognostic tool for a personalized approach to the post-operative evaluation and risk stratification in M-TEER patients.

## 1. Introduction

Mitral regurgitation (MR) is the most prevalent valvular heart disease in the United States, affecting around10% of individuals aged 75 years and older, with its prevalence expected to double by 2030 [1]. Untreated MR leads to reduced quality of life, more frequent heart failure admissions, and increased mortality. Mitral valve (MV) transcatheter edge-to-edge repair (M-TEER) has become a key non-surgical treatment for severe and symptomatic MR, according to the current European and American scientific guidelines [1].

Despite significant advancements in devices and interventions, it is not uncommon for residual MV dysfunction to persist or recur following M-TEER. This is associated with higher rates of hospitalization and adverse cardiac events, significantly impacting a patient’s quality of life [1,2]. Follow-up transthoracic echocardiography (TTE) remains the cornerstone imaging modality for the evaluation of M-TEER function. However, quantitative assessment of residual MV dysfunction can be complicated by valve artifacts and multiple eccentric jets [2,3]. Furthermore, the literature regarding risk stratification following M-TEER has largely relied upon population-based models, failing to account for individual patient heterogeneity, including unique valvular morphology relative to clip placement. Although the prognostic significance of residual MV dysfunction following M-TEER is well recognized, there remains a need for further patient-based studies assessing and validating reliable techniques for long-term surveillance [4].

Previously, multiple TTE-derived parameters have been evaluated for such a purpose. This includes the velocity time integral of the MV and left ventricular outflow tract ratio (VTI_MV/LVOT_), with a cutoff of ≥2.5, which has been shown to be effective for assessing significant MV dysfunction in native valves, scopometric valve replacements, and more recently post-M-TEER [4,5,6]. Furthermore, this VTI_MV/LVOT_ ratio has been demonstrated to have independent prognostic utility in predicting both mortality and major adverse cardiovascular events (MACE) post-M-TEER [4]. However, this tool does not evaluate diastolic dysfunction (DD), which represents a recognized marker of residual MR and can also have prognostic implications in this population. In the setting of native MV dysfunction, an ECG-AI model has been validated to predict the risk of DD and subsequent mortality; however, this model is yet to be evaluated following M-TEER [7].

Given the increasing utility and application of M-TEER in the setting of severe MR, it is paramount to establish more reliable and accurate methods to evaluate residual MV dysfunction and clinical outcomes [8]. In this regard, we sought to determine the prognostic utility of combining the validated VTI_MV/LVOT_ parameter with an ECG-AI model for DD that has been shown to be associated with increased mortality in patients with native MV dysfunction, but has yet to be validated in the M-TEER population.

## 2. Methods

### 2.1. Study Population

All adult patients (≥18 years old) who underwent M-TEER utilizing MitraClip™ (Abbott Laboratories, Chicago, IL, USA) between 2014 and 2021 across three tertiary hospitals in the United States (Mayo Clinic Rochester, Mayo Clinic Arizona, Mayo Clinic Florida) were retrospectively assessed. Patients were required to have at least one comprehensive TTE within three months after M-TEER. In case of multiple available TTEs, the closest one to the procedure was considered. Additionally, each patient was required to have at least one ECG within three months post the procedure, with the closest one to the TTE being selected. Patients without these data were excluded. This study was approved by the Mayo Clinic Institutional Review Board. Baseline characteristics included demographics, comorbidities, etiology of MR, and pre-M-TEER TTE parameters. All data were retrospectively retrieved from electronic medical records (EMRs) using EPIC Hyperspace Production (Epic Systems Corporation, Verona, WI, USA).

### 2.2. Echocardiographic Evaluation

Comprehensive TTE (Philips iE33; Philips Medical Systems; GE Vivid E9, GE Healthcare) within three months after the M-TEER procedure was collected for all the patients in our study. Two-dimensional and Doppler imaging, including both pulsed-wave and continuous-wave Dopplers, were conducted in as with the current American Society of Echocardiography (ASE) guidelines, and the images were reviewed retrospectively [9]. The velocity time integral (VTI) of the MV (VTI_MV_) and left ventricular outflow tract (VTI_LVOT_) were measured manually, and a ratio of VTI_MV_ and VTI_LVOT_ (VTI_MV/LVOT_) was calculated for each patient by dividing VTI_MV_ by VTI_LVOT_ as per our prior report [4]. A high VTI_MV/LVOT_ ratio was defined as ≥2.5 in accordance with previously published literature [5,6].

### 2.3. Electrocardiogram Artificial Intelligence Algorithm

All ECGs within three months post-procedure were retrieved and analyzed by a previously validated AI-predictive algorithm embedded in the institutional EMR, from which predictive risk scores were collected for DD, ranging from 0–3 with increasing diastolic grading [10,11]. This model was trained in 98,736 patients and subsequently validated and tested in over 120,000 patients. The areas under the curve diagnostic accuracy of this tool to detect DD grades were 0.85 for grade ≥1, 0.91 for grade ≥2, and 0.94 for grade 3. For the detection of DD grade ≥2, the sensitivity was 83.2% and the specificity was 82.9%. In our cohort, an ECG-AI score for DD for each patient was calculated. The cohort was grouped into those with low risk for DD vs. high risk for DD. Low risk for DD was defined as an ECG-AI score of 0 or 1, and high risk for DD was defined as an ECG-AI score of 2 or 3.

### 2.4. Outcomes

Clinical outcomes were retrospectively collected from the EMR up to one year following M-TEER and included all-cause mortality, major adverse cardiac events (MACE: composite of heart failure admission, stroke, or myocardial infarction), and MV re-intervention. VTI_MV/LVOT_ on TTEs was combined with a concurrent ECG-AI risk score for DD to create three groups: normal VTI_MV/LVOT_ (<2.5) and ECG-AI score (≤1), abnormal VTI_MV/LVOT_ (≥2.5) or ECG-AI score (>1), and both abnormal VTI_MV/LVOT_ (≥2.5) and ECG-AI score (>1). Comparisons of clinical outcomes were made between the three groups.

### 2.5. Statistical Analysis

Statistical comparisons of baseline characteristic data between the groups were conducted using *t*-test or non-parametric tests for continuous variables (according to distribution) and chi-square test or Fisher’s Exact Test for categorical variables. Continuous variables were summarized as mean and standard deviation (mean ± SD) or median with interquartile range (IQR) according to distribution, while categorical variables were presented as frequencies with percentages.

Survival free from progression was compared between the groups using Kaplan–Meier curves with pairwise Log Rank testing and Cox regression analyses. Multivariable analyses were adjusted for age, sex, hypertension, type II diabetes mellitus, history of ischemic heart disease, history of atrial fibrillation, and type of MR (primary or secondary). Time zero was set at the time of M-TEER, and patients were censored by clinical event, death, or at one-year post-M-TEER. Results were reported as hazard ratio (HR) with corresponding 95% confidence intervals (95% CIs). The Proportional Hazard Assumption was evaluated in every model for the variables of interest using Schoenfeld Residuals plots against time, as well as the Grambsch and Therneau test. The Variance Inflation Factor was used for detecting multicollinearity in the Cox regression models. To assess whether the addition of ECG-AI to the validated VTI_MV/LVOT_ improved the classification of patients’ 1-year mortality/MACE risk, we computed the category-free Net Reclassification Improvement (NRI). We compared the model including VTI_MV/LVOT_ alone with the additive model including both VTI_MV/LVOT_ and ECG-AI. The NRI was calculated for events (NRI+) and non-events (NRI–) separately, as well as the overall NRI.

Two-tailed *p* values < 0.05 were considered statistically significant for all analyses. Statistical analyses for the comparison of baseline characteristics as well as survival analyses were conducted using IBM SPSS Statistics (v 28.0, IBM Corporation, Armonk, NY, USA) and RStudio 2025.05.1 Build 513 © 2009–2025 Posit Software (including survival, car, nricens packages).

## 3. Results

Overall, 250 patients were included, with baseline characteristics presented in Table 1. There were 37 (14.8%) patients with normal VTI_MV/LVOT_ + ECG-AI score, 143 (57.2%) patients with one abnormal parameter, and 70 (28.0%) patients with both abnormal parameters. There were no significant differences between the three groups regarding age and sex. Patients with both abnormal parameters had a higher incidence of atrial fibrillation/flutter at baseline, with no other differences in cardiovascular comorbidities. There was also no significant difference in the rate of prior cardiac valve interventions.

**Figure 1:**
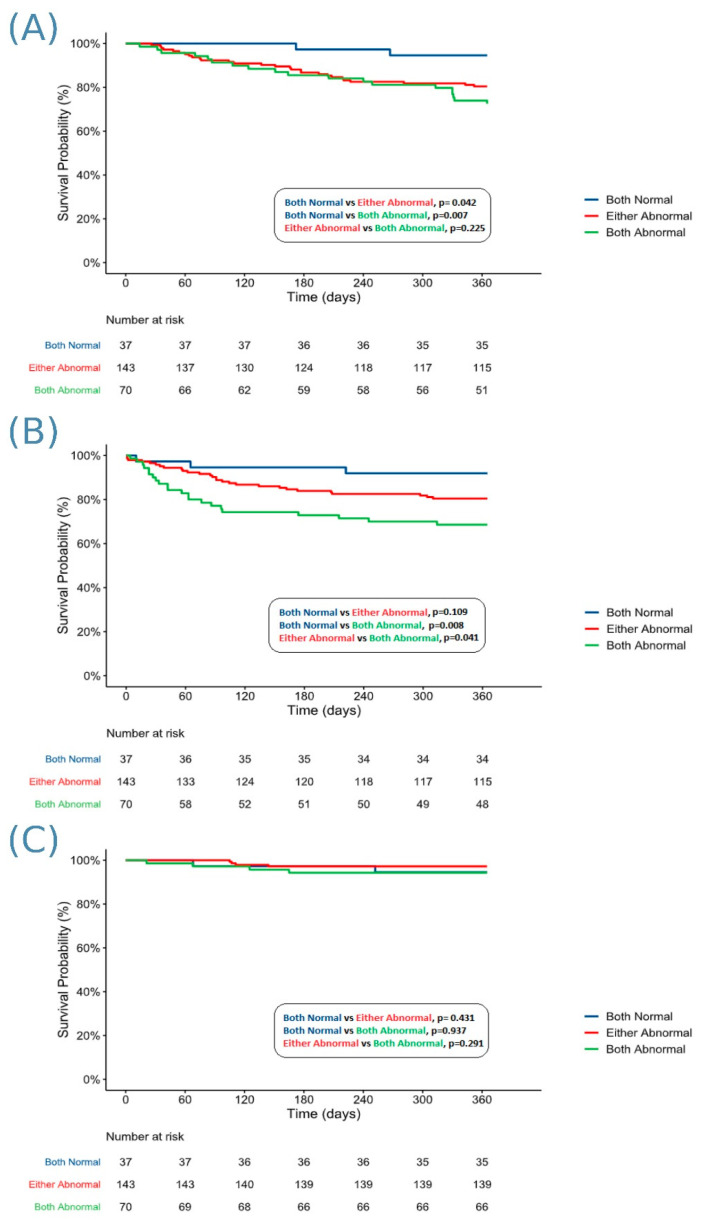
Kaplan–Meier survival curves comparing the survival free from (**A**) mortality, (**B**) MACE, and (**C**) MV reintervention between patients who had both the parameters normal (VTI_MV/LVOT_ < 2.5, ECG-AI score ≤ 1), either parameter abnormal (VTI_MV/LVOT_ ≥ 2.5, ECG-AI score > 1), or both parameters abnormal (VTI_MV/LVOT_ ≥ 2.5 and ECG-AI score > 1).

On Kaplan–Meier analysis of mortality at one year following M-TEER, patients with both abnormal VTI_MV/LVOT_ and ECG-AI or one abnormal parameter had significantly lower survival compared to patients with both normal parameters; KM estimated survival 72.5% and 80.4% compared to 94.6% (log rank *p* = 0.007 and *p* = 0.042, respectively) (Figure 1A). In multivariable analysis, only patients with both abnormal parameters had a significantly higher risk of mortality, compared to patients with both normal parameters (aHR 4.56, 95%CI 1.04–19.89, *p* = 0.044, Table 2). No significant differences were found in the multivariable analysis when comparing one-year mortality between patients with one abnormal parameter versus both abnormal; however, an NRI of 0.20 was observed, suggesting an improvement in individual risk prediction after the addition of the ECG-AI tool to the VTI score. There were 53 MACEs in the overall cohort, with the majority being heart failure re-admissions (84.9%). In Kaplan–Meier analysis, patients with both elevated VTI_MV/LVOT_ and ECG-AI score had lower survival probability free from MACE compared to patients with one abnormal parameter (68.6% vs. 80.4%, log rank *p* = 0.041) and patients with both normal parameters (68.6% vs. 91.9%, log rank *p* = 0.008) (Figure 1B). There was no significant difference between the patients with one abnormal or both normal parameters (log rank *p* = 0.109). This result persisted on multivariable analysis, where patients with both elevated VTI_MV/LVOT_ and ECG-AI score had a higher risk of MACEs compared to patients with both normal parameters (aHR 3.72, 95%CI 1.09–12.72, *p* = 0.037) and compared to one abnormal parameter (aHR 1.81, 95%CI 1.03–3.20, *p* = 0.041, Table 2). Furthermore, after the addition of the ECG-AI tool to the VTI_MV/LVOT_, an NRI of 0.19 was detected, confirming the enhanced individual patient risk prediction after the addition of the ECG-AI tool.

Kaplan–Meier analysis showed no significant differences between patients with both abnormal parameters compared to patients with either one abnormal or both normal parameters in rates of reintervention on the MV (log rank *p* = 0.291 and *p* = 0.937, respectively) (Figure 1C). Furthermore, a combined elevated VTI_MV/LVOT_ and ECG-AI score was not associated with increased risk of reintervention on the MV in the multivariable analysis (Table 2).

## 4. Discussion

In this study, we have demonstrated that a combination of abnormal ECG-AI score for DD (>1) and a VTI _MV/LVOT_ ratio ≥ 2.5 was independently associated with worse prognosis for both one-year mortality and MACE in patients post-M-TEER. As previously seen with VTI_MV/LVOT_ score alone, there was no association between the combined score and re-intervention of the MV. Moreover, the combination of the two parameters (echocardiography + ECG-AI) slightly outperformed the use of the validated VTI_MV/LVOT_, especially for the prediction of MACE following M-TEER, and showed modest but positive improvement of the risk classification as suggested by NRI. Ultimately, the integration of TTE markers with ECG-AI modeling in this study holds promise for the early identification of high-risk individuals as well as providing a framework for individualized procedural planning, surveillance strategies, and post-procedure management.

The increasing incidence of M-TEER procedures has raised concerns about post-procedure durability and complications [12]. Residual MR and stenosis after M-TEER have been linked to worse survival rates and increased heart failure events, but their assessment is extremely complex in the post-M-TEER setting [12,13]. Left ventricular DD can also reflect MV dysfunction and could impact the prognosis of patients with MV disease; however, its quantification using echocardiography is challenging and variable among different operators. Considering this, and with the goal of better understanding prognostication following M-TEER, our study is the first to evaluate the prognostic value of integrating the ECG-AI score for DD and VTI_MV/LVOT_ ratio in M-TEER patients.

Significant MR causes left atrial dilation and dysfunction due to elevated left ventricular filling pressure and increased systolic blood volume entering the left atrium [7,11]. This condition alters early diastolic mitral inflow velocity, disrupts pulmonary vein blood flow, and raises pulmonary arterial pressures, complicating the echocardiographic diagnosis of left ventricular DD. Given that worsening MR increases left ventricular filling pressure and disrupts diastolic function, ECG-AI could play a significant role in prognostic stratification for individuals with significant MV dysfunction post-M-TEER. Tsaban et al. demonstrated that ECG-AI can grade diastolic function in patients with moderate to severe native MR and that increased DD correlates with higher mortality risk, even after adjusting for confounders. In his study, ECG-AI DD grading added value to traditional echocardiographic grading in prognostic stratification for patients with significant MR [7,11].

Our study demonstrates the additive role of ECG-AI DD grading in predicting the risk of patients post-M-TEER procedure. Previously, AI models have been evaluated for predicting outcomes in TEER patients, which included several metabolic factors showing better mortality prediction compared to traditional cardiovascular risk factors and previously reported risk scores. However, the model did not incorporate ECG-AI tools or any echocardiographic measurements [14].

Traditional methods for assessing MR post-M-TEER, such as proximal isovelocity surface area (PISA), are often not feasible, and alternative methods, such as assessing vena contracta area (VCA) via 3D Doppler TEE, have shown some promise but are not thoroughly validated. Non-invasive methods like cardiac MRI have been suggested but have limitations, including technical issues and patient incompatibility, highlighting the challenges of MV dysfunction surveillance [4]. The easily attainable Doppler VTI_MV/LVOT_ ratio’s effectiveness in assessing MV dysfunction after surgical interventions has been discussed in a previous study, which suggested a VTI_MV/LVOT_ cutoff of ≥ 2.5 for significant MR, which was later confirmed by other studies [5,6]. Scalia et al. demonstrated that the VTI _MV/LVOT_ ratio is useful in evaluating residual MV dysfunction in post-TEER patients, showing a significant correlation with residual MR and MV gradients. The study found that the VTI_MV/LVOT_ ratio could predict all-cause mortality, heart failure readmission, and MACE within a year post-procedure, and that high VTI_MV/LVOT_ values indicated a higher risk of adverse outcomes [4].

Our study further demonstrates the additive role of ECG-AI for DD grading in predicting high-risk patients post-M-TEER. The novelty of our study lies in combining these two parameters, demonstrating prognostic utility and reclassification improvement compared to both normal parameters. From a practical standpoint, the potential utility of this combined tool lies in its ability to incorporate personalized risk modeling into routine clinical pathways post-M-TEER. In this way, it may facilitate early and shared decision making and ultimately reduce adverse outcomes in high-risk patients.

### Limitations

The primary limitations of this study stem from its retrospective nature, which may have allowed for confounders beyond our control. The specific ECG-AI model evaluated in the present study was developed using retrospective data in the general population, collected exclusively at a major tertiary medical center in the United States. As such, this is the first study to assess its validity and utility in the M-TEER population. Given this, as well as the small sample size, the number of covariates and event rates included in the multivariable Cox Regression model, there may be some limitations to the generalizability of our results. The modest values of the NRI suggest that adding ECG-AI to the VTI model modestly improved risk classification; therefore, these results should be interpreted alongside other model performance measures and clinical impact. Finally, this study reported the clinical outcomes at one-year post-M-TEER, whereby future studies with extended follow-up may further enhance the clinical relevance and external validation of this assessment tool.

## 5. Conclusions

Combined abnormal VTI_MV/LVOT_ (≥2.5) on TTE and ECG-AI for DD grade (>1) was associated with worse outcomes at one-year follow-up in patients post-M-TEER in our cohort. With future external validation, these easy-to-apply scores may assist in simplifying the post-operative evaluation and risk stratification, providing a personalized approach to post-M-TEER assessment.

## Figures and Tables

**Table 1:** Comparison of baseline characteristics and echocardiographic measurements between the three groups according to VTI_MV/LVOT_ cutoff of ≥2.5 and ECG-AI score ≤ 1 *.

Baseline Characteristics	VTI_MV/LVOT_ < 2.5 & ECG-AI Score ≤ 1 “Both Normal” (*n* = 37)	VTI_MV/LVOT_ ≥ 2.5 or ECG-AI Score > 1 “One Abnormal” (*n* = 143)	VTI_MV/LVOT_ ≥ 2.5 & ECG-AI Score > 1 “Both Abnormal” (*n* = 70)	Overall Patient Cohort (*n* = 250)	*p* Value
**Age at time of M-TEER, years**	79.7 (74.5, 83.5)	79.4 (71.8, 84.6)	81.5 (73.5, 84.5)	79.5 (73.1, 84.6)	0.677
**Sex, male**	23 (62.2)	95 (66.4)	48 (68.6)	166 (66.4)	0.800
**Hypertension**	29 (78.4)	121 (84.6)	55 (78.6)	205 (82.0)	0.461
**Diabetes**	11 (29.7)	42 (29.4)	25 (35.7)	78 (31.2)	0.630
**Atrial fibrillation/flutter**	19 (51.4)	104 (72.7)	52 (74.3)	175 (70.0)	0.027
**MR etiology**					
**MR Primary**	31 (83.8)	90 (62.9)	44 (62.9)	165 (66.0)	0.047
**MR Secondary**	6 (16.2)	53 (37.1)	26 (37.1)	85 (34.0)	0.047
**Prior ischemic heart disease**	7 (18.9)	47 (32.9)	26 (37.1)	80 (32.0)	0.149
**Prior valvular intervention**	4 (10.8)	18 (12.6)	12 (17.1)	34 (13.6)	0.572
**Prior aortic valve intervention**	3 (8.1)	12 (8.4)	9 (12.9)	24 (9.6)	0.551
**Prior tricuspid valve intervention**	2 (5.4)	4 (2.8)	4 (5.7)	10 (4.0)	0.531
**Prior mitral valve intervention**	0 (0.0)	6 (4.2)	3 (4.3)	9 (3.6)	0.444
**Pre M-TEER Echocardiographic parameters**					
**Ejection fraction, %**	60.0 (55.0, 65.0)	53.0 (35.0, 60.0)	50.0 (35.5, 60.0)	55.0 (39.0, 60.0)	<0.001
**Right ventricular systolic pressure, mmHg**	41.0 (33.0, 47.0)	50.0 (41.0, 59.0)	51.0 (41.0, 63.0)	49.0 (40.5, 59.0)	<0.001
**LV dimension (d), mm**	54.0 (50.0, 58.0)	56.0 (52.0, 62.0)	56.0 (52.3, 63.5)	56.0 (51.0, 61.0)	0.064
**LV dimension (s), mm**	35.0 (29.5, 38.5)	38.0 (33.0, 48.0)	40.0 (33.0, 50.0)	38.0 (32.3, 47.8)	0.007

Abbreviations: ECG-AI: electrocardiogram artificial intelligence; IQR: interquartile range; LV: left ventricle; MR: mitral regurgitation; M-TEER: mitral valve transcatheter edge-to-edge repair; VTI_MV/LVOT_: velocity time integral of the mitral valve and left ventricular outflow tract ratio. * Continuous variables were reported as median (IQR), and categorical variables were reported as *n* (%).

**Table 2:** Results of multivariable Cox Regression analyses assessing the association of three groups according to VTI_MV/LVOT_ cutoff of ≥2.5 and ECG-AI score > 1 with study outcomes.

Outcomes	VTI_MV/LVOT_ < 2.5 & ECG-AI ≤ 1 vs. VTI_MV/LVOT_ ≥ 2.5 or ECG-AI > 1		VTI_MV/LVOT_ < 2.5 & ECG-AI ≤ 1 vs. VTI_MV/LVOT_ ≥ 2.5 & ECG-AI > 1		VTI_MV/LVOT_ ≥ 2.5 or ECG-AI > 1 vs. VTI_MV/LVOT_ ≥ 2.5 & ECG-AI > 1	
	Adjusted HR [95% CI]	*p* value	Adjusted HR [95% CI]	*p* value	Adjusted HR [95% CI]	*p* value
**Mortality**	3.26 [0.77–13.89]	0.110	4.56 [1.04–19.89]	**0.044**	1.39 [0.77–2.52]	0.267
**MACE**	2.05 [0.61–6.89]	0.245	3.72 [1.09–12.72]	**0.037**	1.81 [1.03–3.20]	**0.041**
**MV reintervention**	0.63 [0.11–3.58]	0.601	1.44 [0.24–8.50]	0.687		

Abbreviations: CI: confidence interval; ECG-AI: electrocardiogram artificial intelligence; HR: hazard ratio; MACE: major adverse cardiovascular events; MV: mitral valve; VTI_MV/LVOT_: velocity time integral of the mitral valve and left ventricular outflow tract ratio.

## Data Availability

Data may be made available at reasonable request to the corresponding author.
